# Study of Ultrastructural Abnormalities in the Renal Cells of *Cyprinus carpio* Induced by Toxicants

**DOI:** 10.3390/toxics10040177

**Published:** 2022-04-02

**Authors:** Sumayya Nazir, Md. Niamat Ali, Javeed Ahmad Tantray, Irfan Akram Baba, Arizo Jan, Simona Mariana Popescu, Bilal Ahamad Paray, Aneela Gulnaz

**Affiliations:** 1 Department of Zoology, University of Kashmir, Srinagar 190006, J&K, India; 2 Cytogenetics and Molecular Biology Research Laboratory, Centre of Research for Development (CORD), University of Kashmir, Srinagar 190006, J&K, India; 3 Department of Zoology, Central University of Kashmir, Ganderbal 191201, J&K, India; javeedh3@gmail.com; 4 Department of Livestock Production and Management (LPM), Faculty of Veterinary Sciences and Animal Husbandry, Sher-e-Kashmir University of Agricultural Sciences and Technology (SKUAST-K), Shalimar, Srinagar 190025, J&K, India; irfanvet@gmail.com; 5 Division of Fisheries Resource Management, Faculty of Fisheries, Sher-e-Kashmir University of Agricultural Sciences and Technology (SKUAST-K), Shalimar, Srinagar 190025, J&K, India; arzujaan65@gmail.com; 6 Department of Biology and Environmental Engineering, University of Craiova, 13, A.I. Cuza, 200585 Craiova, Romania; popescu_simona3@yahoo.com; 7 Department of Zoology, College of Science, King Saud University, P.O. Box 2455, Riyadh 11451, Saudi Arabia; bparay@ksu.edu.sa; 8 College of Pharmacy, Woosuk University, Wanju-gun 55338, Korea; draneela@woosuk.ac.kr

**Keywords:** Phorate, dimethoate, TEM, renal cells, ultrastructure, toxicants

## Abstract

Transmission Electron Microscopic (TEM) assessments were performed on the renal cells of common carp *Cyprinus carpio* to observe the deleterious effects of two organophosphate insecticides, Phorate and Dimethoate. Pesticides such as Phorate and Dimethoate often pollute aquatic systems where they may negatively impact fish, but so far, the ultrastructural toxicity of these pesticides remains poorly understood. Here, we use Transmission Electron Microscopy (TEM) to determine how acute exposure to sublethal concentrations of these two pesticides may affect the renal cells of common carp *Cyprinus carpio*. For each insecticide, the fish were divided in four experimental conditions: a control and three different exposure concentrations of the pesticide. The Phorate treated fish were exposed to three sublethal concentrations of 0.2 mg/L, 0.4 mg/L, 0.6 mg/L for a duration of 24, 48 & 72 h. The dimethoate treated fish were exposed to three sublethal concentrations of 0.005 mL/L, 0.01 mL/L, 0.015 mL/L for a duration of 24, 48 and 72 h. The two-dimensional transmission electron microscopy revealed ultrastructural abnormalities in the treated fish renal cells when exposed to two toxicants including deformation in the glomerulus, vacuolization of cytoplasm, degenerative nucleus and damaged mitochondria. Furthermore, the ultrastructural abnormalities were more prominent with the increase in the concentrations of both the insecticides and also with their exposure period. Overall, these results provide important baseline data on the ultrastructural toxicity of Phorate and Dimethoate and will allow important follow-up studies to further elucidate the underlying cellular mechanisms of pesticide toxicity in wildlife.

## 1. Introduction

Pesticides have been used successfully to improve agricultural productivity and meet the increasing demand for food, but they often end up in the natural environment where they may impact non-target organisms. Pesticides can act on organisms other than pest species. This is of particular concern in developing countries, where increasing intensification of agriculture leads to high pesticide use [1]. Pesticides cover a wide range of substances including insecticides, acaricides, fungicides, molluscicides, herbicides, nematocides and rodenticides. Pesticides are widely used in agriculture to control insects, nematodes, fungi, etc. that affect food and other crops. These are easy to apply, being cost effective and, most importantly, they are readily available practical means of pest control. On the flip side, pesticides may have unwanted effects on the natural environment as they can enter through various routes including direct application, spray drift, atmospheric deposition and surface runoff. Among the different types of pesticides, insecticides are often used. Depending upon the mode of action, insecticides have been classified by the Insecticide Resistance Action committee (IRAC, 2017) into various categories, viz., botanicals (nicotine, rotenone, pyrethrum, etc.), organochlorines (e.g., DDT), organophosphates (Phorate, Dimethoate), carbamates, pyrethroids etc [2]. There are many pathways by which pesticides leave their sites of application and are distributed throughout the aquatic ecosystem. Different concentrations of the pesticides are present in many types of wastewater and several studies have revealed them to be toxic to aquatic organisms including fish [3,4]. Fish are sensitive to polluted water. Hence, fish have long being used to monitor the quality of the aquatic environment and fish histology is increasingly being used as an indicator of environmental stress [5,6].

Phorate is a systemic and broad spectrum organophosphorus (OP) insecticide, commonly used in agriculture to control sucking and chewing insecticides, leaf hoppers and mites. It is also used in pine forests and on root and field crops including corn, cotton, coffee and some ornamental plants and bulbs [7,8]. Phorate is primarily formulated as granules to be applied at planting in a band or directly to the seed furrow. In biota, it inhibits acetylcholinesterase activity by phosphorylating the serine hydroxyl group in the substrate binding domain, which results in the accumulation of acetylcholine and induces neurotoxicity. Though its use has been strictly restricted by the United States Environmental Protection Agency (USEPA), it is still being used in several countries such as India, China, Italy and Egypt [9].

Dimethoate is a systemic organophosphate insecticide used on a large variety of field grown agricultural crops, tree crops, and ornamentals. Dimethoate was first registered in 1962 in the US and later its use for non-agricultural practices (e.g., domestic purposes) was banned as of 2000. It is available as Rogor and like other organophosphates it acts as an acetylcholinesterase inhibitor and works as a nerve poison at synapses of neuromuscular junctions, which is evident by abnormal body movements and jerks [10,11,12]. Various studies on the toxicity of Phorate on aquatic organisms, especially fish, have been carried out, confirming its toxicity and genotoxic role [13,14,15]. Histopathological toxicity of brain of *Cyprinus carpio* exposed to Phorate was also reported by Lakshmaiah, (2017) [16]. Lakshmaiah (2016) reported acute toxicity of Phorate on succinate dehydrogenase enzyme activity, which is an important enzyme for Krebs Cycle [17]. Additionally, its genotoxicity potential was reported by Saquib et al., (2012) in male wistar rats and in human amniotic epithelial (WISH) cells [7]. Toxicity of dimethoate in fish fauna has also been reported by many researchers [12,18]. Demet and Canan (2011) reported toxic effects of Dimethoate on hematological, biochemical and behavioural patterns in *Oncorhynchus mykiss* exposed to sublethal concentrations of 0.0735, 0.3675, and 0.7350 mg/L for 5, 15, and 30 days [19]. Ganeshwade (2012) reported biochemical changes in the gills of freshwater fish *Puntius ticto* when exposed to lethal (5.012 ppm) and two sublethal (2.506 and 1.253 ppm) concentrations of Dimethoate for 96 h and 60 days, respectively [20]. Histopathological studies were reported in the kidney of *Cyprinus carpio* after exposure to dimethoate (EC 30%) by Singh (2012) [21]. Binukumari and Vasanthi (2013) observed the toxic effect of the Dimethoate 30% EC on protein metabolism of *Labeo rohita,* when exposed to a concentration of 0.398 ppm for 24, 48 and 72 h, respectively [22]. Singh (2013) exposed common carp, *Cyprinus* to dimethoate 0.40 mg/L for short-term exposure of 96 h and reported toxic effects on its liver [23]. Singh (2014) reported toxic effects of Dimethoate (EC 30%) on gill morphology, oxygen consumption and serum electrolyte levels of common carp, *Cyprinus Carpio.* The fish were exposed to a sub lethal concentration of 0.96 mg/L (60% of 96 h LC50) of dimethoate at a 24, 48 and 96 h exposure duration [24]. Singh (2017) reported testicular toxicity of *Cyprinus carpio* when exposed to dimethoate at 0.96 mg/L and 0.48 mg/L, respectively in a short-term (96 h) and long-term study (36 days) [25].

As is evident, the toxic effects of these two organophosphates have been reported but no work has been reported on their potential role as cytotoxins, i.e., their role to cause ultrastructural abnormalities in fish cells. So, the present study has been designed to investigate the role of two organophosphate insecticides: Phorate and dimethoate, for their potential to damage cells in an in vivo system and contribute to acute toxicity. Hence, an investigation resulting from cellular events leading to cytotoxicity is proposed. Teleost fish have proved to be good models to evaluate the genotoxicity and effects of pollutants such as insecticides on animals, as their biochemical responses are similar to those of mammals and other vertebrates [14,26,27,28]. The advantage of using fish models includes the facility by which Teleostei, especially the small species, can be maintained and handled inside the laboratory under experimental conditions of toxic exposure [29]. Fish frequently respond to chemical exposure as superior vertebrates, which validate this model to study potential teratogenic and carcinogenic compounds in humans [30]. Common carp *Cyprinus carpio* is a robust fish which can tolerate a wide range of temperatures and is adaptable to varying environmental conditions. It is an economically important fish due to its nutritional value. Thus, it serves as an important vehicle for contamination.

The main objective of the present study was to observe Phorate and Dimethoate induced ultrastructural abnormality in the renal cells of common carp, *Cyprinus carpio* (as the kidney is not only the excretory organ but also functions as osmoregulatory organ of the fish). It will provide baseline data about the ultrastructural toxicity of these insecticides, which are widely used and finally reaches into our precious water bodies, thereby proving hazardous to both aquatic fauna (especially fish), as well as to its consumers. The present study was initiated to understand the acute toxicity of organophosphate insecticide exposure which could make a potential contribution towards the identification of the environmental toxicants. Such an acute toxicity testing would measure the adverse effects that occur within a short period of time. Thus, such information would be fruitful to serve as a basis for hazard classification and will provide information regarding the target tissue/organ toxicity by different doses of the toxicants.

## 2. Materials and Methods

The fish species *Cyprinus carpio* L. (family: Cyprinidae) were chosen for the present study. Young specimens of *Cyprinus carpio communis* (age: <1 year; weight 30–40 g; length 10–12 cm) were used. The healthy young adult specimens of *Cyprinus carpio communis* were collected from the Dal Lake (34°5′–34°6′ N latitude and 74°8′–74°9′ E longitudes) using a net and hand-picking method, and then these collected fish were transported to a laboratory in a specially-designed container with oxygen supply. After collection, fish were acclimatized for 15 days in 60 L artificially aerated glass aquaria (5 fish each) containing dechlorinated tap water for 12/12 natural photoperiod (pH 7.6–8.4, temperature 25 ± 3). During acclimatization, fish were fed a commercial diet (Feed Royal^®^, Maa Agro Foods, Visakhapatnam, Andhra Pradesh, India). In order to avoid ammonia accumulation, the water in these aquariums were changed daily with dechlorinated tap water. During the test period, no feed was given to keep the insecticide concentrations constant throughout the test period of 72 h [18,19]. Water quality of the test solution was determined according to standard procedures [31]. The control fish were kept in experimental water without adding these insecticides, keeping all other conditions constant.

### 2.1. Test Chemicals, Selection and Dosage of the Insecticides

The pesticides used for the present study were two widely used organophosphorus insecticides: Phorate and Dimethoate. Commercial grade formulations of insecticides were used because only commercial preparations are used in agriculture. The commercial grade of Phorate was obtained from agro farmers, Court Road, Srinagar manufactured by Sikkim Pesticide Industries. Rogor^®^ (30% dimethoate) was obtained from Big Farmers, Jahangir Chowk near Budshah bridge manufactured by Cheminova, A/S Denmark. Dimethoate is fairly stable than phorate in aqueous solutions (Table 1).

On the basis of the literature data (LC50 values for each insecticide), three sublethal concentrations of these insecticides were selected for the experiment, 0.2 mg/L 0.4 mg/L and 0.6 mg/L for Phorate and 0.005 mL/L, 0.01 mL/L, 0.015 mL/L for Dimethoate.

### 2.2. Experimental Design

In order to assess the ultrastructural abnormalities induced by the Phorate and Dimethoate in the fish renal cells, the fish for each insecticide were placed in four experimental conditions: a control and three different exposure concentrations. Based on the selected doses of each insecticide and the time of exposure. All groups had an equivalent no. of fishes, i.e., 5 fish per group in a glass aquarium. After completion of the exposure period of 72 h, the fish were euthanized, and the kidneys were removed to assess ultrastructural abnormalities using transmission electron microscopy (TEM). It was briefly washed in 0.09% normal saline soon after biopsy of the animal in order to remove the associated mucus or blood from it. The kidney was cut into small pieces of 1 × 1 mm. Then, these tissues were fixed in a mixture of 2% paraformaldehyde and 2.5% glutaraldehyde in 0.1 M phosphate buffer for 8–12 h at 4 °C. Then, the tissues were washed in buffer three times, each for one hour duration at 4 °C. The tissues were post fixed in 1% osmium tetroxide for 1 h at 4 °C. The tissues were then dehydrated through an acetone series (30%, 50%, 70%, 90% and 100%) for 15 min each. The dehydrated tissues were then cleared off the acetone by propylene oxide for 30 min and then infiltrated and embedded in a liquid resin epoxy. After embedding, the resin blocks were then thin-sectioned and these ultrathin sections (70 nm) were placed on metal grids and stained with electron dense stains, with uranyl acetate and lead citrate. These samples were then examined and photographed for ultrastructural changes by TEM (TECNAI 200 kV, FEI/Phillips, Hillsboro, OR, USA) at AIIMS, New Delhi for observations and photography.

## 3. Results

**Figure 1:**
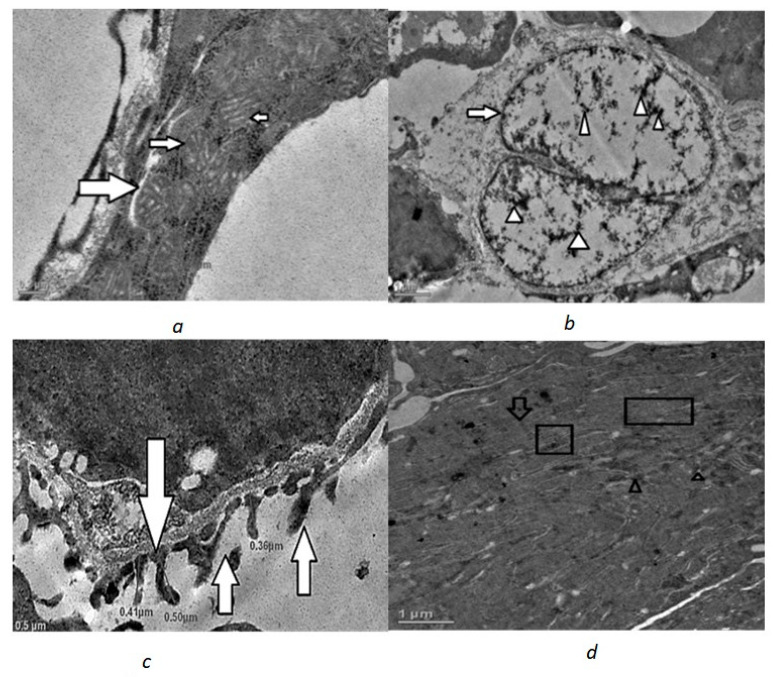
Transmission electron micrographs of control renal cells with intact normal architecture of cellular organelles: (**a**) normal mitochondria with distinct cristae (white filled arrow) (scale 0.2 µm); (**b**) nucleus, though binucleate (white filled arrow) but with uniformly distributed chromatin (white arrow head) (scale 1 µm); (**c**) pedicel with infiltration slits (white filled arrow) (scale 0.5 µm); (**d**) stacks of endoplasmic reticulum (boxed), mitochondria (arrow) and lysosomes (arrow head) (scale 1 µm).

Kidney samples from the control fish showed normal structural features. The nucleus was oval in shape with heterochromatin evenly distributed throughout the nucleoplasm and in a binucleate condition. The heterochromatin was uniformly distributed (Figure 1b). The mitochondria were tubular with distinct cristae (Figure 1a); stacks of rough endoplasmic reticulum were easily seen around the nucleus and mitochondria (Figure 1d) lysosomes were also seen. The basement membrane also had normal nuclei. Additionally, the pedicels which arise from the specialised epithelial cells (podocytes) were easily seen forming narrow infiltration slits in the glomerulus (Figure 1c).

**Figure 2:**
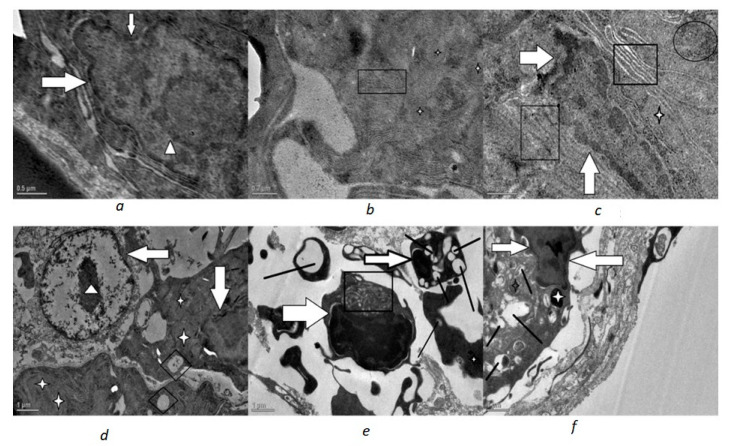
Transmission electron micrographs of 0.2 mg/L Phorate exposed renal cells exposed for 72 h revealing degenerative changes: (**a**) deformed nucleus (white filled arrow) and condensed heterochromatin (arrow head) (scale 0.5 µm; (**b**) degenerated endoplasmic reticulum (□) and condensed degenerated mitochondria(white star) (scale 0.2 µm). (**c**) more degenerative and deformed nucleus (white arrow head), dilated and deformed ER (□), increased lysosomes (circled) and deformed mitochondria (white star) (scale 0.5 µm); (**d**) deformed nucleus (white filled arrow) and electron dense condensed chromatin (white arrow head) and vacuole (boxed ◊) (**e**) showing severe electron dense deposits in the deformed nucleus (white filled arrow) with whorl of degenerated ER (□), severe cytoplasmic vacuolation (thick black line) and deformed mitochondria (star) (scale 1 µm); (**f**) deformed nucleus (white filled arrow), degenerated mitochondria (star), severe vacuolation (thick black line) (scale 1 µm).

**Figure 3:**
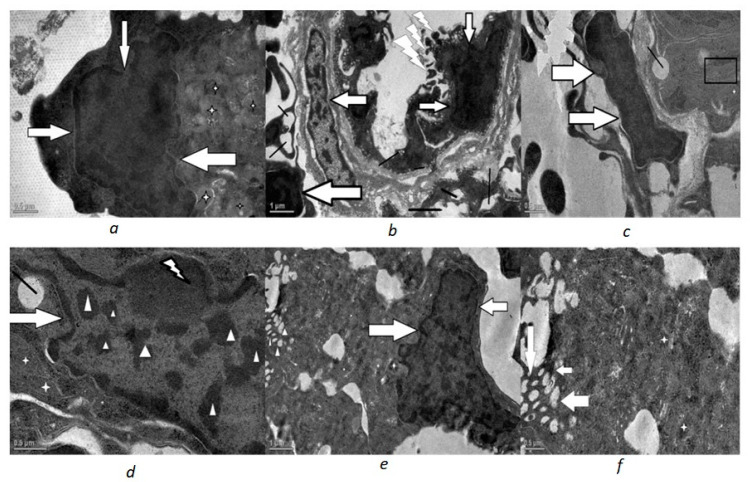
Transmission electron micrographs of 0.6 mg/L Phorate exposed renal cells exposed for 72 h: (**a**) deformed nucleus (white head arrow) and other structures not distinct, mitochondria (star) (scale 1 µm); (**b**) electron dense deposits in deformed nucleus (white filled arrow), deformed fused swollen pedicels (flashed white arrow) and extreme vacuole presence(thick black line) (scale 1 µm). (**c**,**d**) transmission electron micrographs of 0.005 mL/L Dimethoate exposed renal cells exposed for 72 h: (**c**) degenerated nucleus with electron dense deposits throughout nucleoplasm (white filled arrow); condensed degenerated mitochondria (star), vacuole (thick black line) and curved degenerated ER (□) (scale 0.5 µm); (**d**) deformed nucleus (white filled arrow) with condensed electron dense chromatin (arrow head) and peripheral nucleolus (flashed white arrow), presence of vacuoles(thick black line) (scale 0.5 µm) (**e**,**f**) transmission electron micrographs of 0.01 mL/L Dimethoate exposed renal cells exposed for 72 h: (**e**) less distinct cytoplasmic structures-deformed nucleus (white filled arrow), degenerated mitochondria (star), severe cytoplasmic vacuoles (white arrow head) (scale 1 µm); (**f**) extreme vacuolation (white filled arrow) and electron dense degenerated mitochondria (star) (scale 0.5 µm).

**Figure 4:**
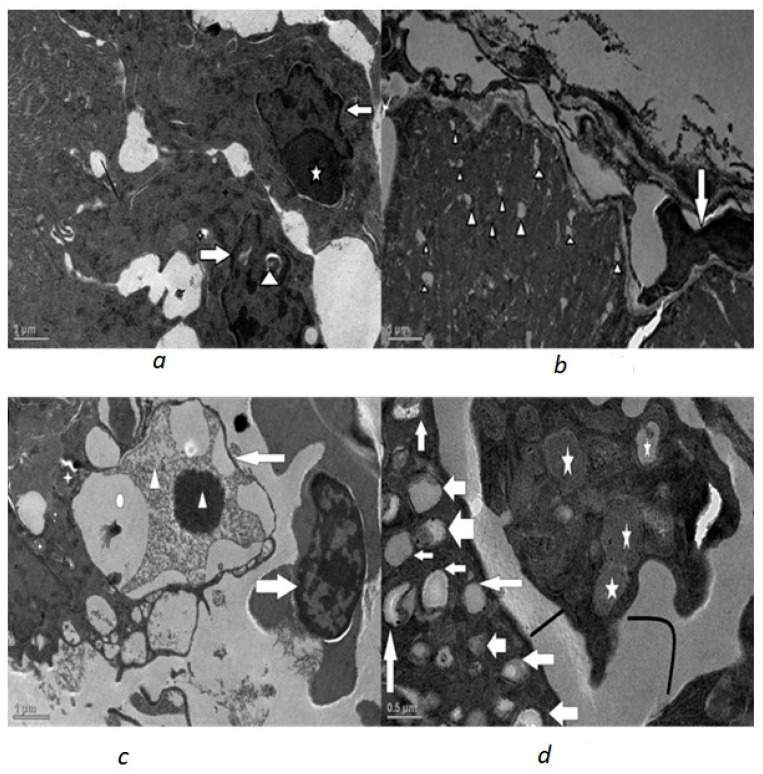
Transmission electron micrographs of 0.015 mL/L Dimethoate exposed renal cells exposed for 72 h: (**a**) highly deformed nucleus (white filled arrow) with apoptotic body (big star), cytoplasmic vacuole (thick black line), vacuolated mitochondria (small star) (scale 1 µm); (**b**) severe cytoplasmic vacuolization (star head), deformed nucleus(white filled arrow) (scale 1 µm); (**c**) deformed nucleus (white filled arrow) with nuclear vacuoles (O) and condensed chromatin (arrow head) and nucleolus (scale 1 µm); (**d**) highly deformed mitochondria (star), increased intercellular space (thick straight and curved line), and extreme cytoplasmic vacuolation (white filled arrow) (scale 0.5 µm).

In both the Phorate- and Dimethoate-treated fish, the ultrastructural changes were evident (Figure 2, Figure 3 and Figure 4). Marked changes were observed in the shape of nuclei in which the nuclear envelope showed shrinkage and, hence, an irregular outline. The mitochondria displayed degenerative changes with condensed and rounded appearance, having disorganized lamellar cristae. The endoplasmic reticulum was also disorganized and fragmented and less in number. Vacuolization was also very much evident in the cytoplasm of the treated fish.

In the glomerulus of Phorate-treated fish, most of the podocytes exhibited swollen, fused and short pedicel with a significant increase in filtration slit width. These degenerative changes became more and more evident with the increase in the concentration of the Phorate for the exposure time period of 72 h. With the increase in dose, the number of mitochondria increased with distinct degenerative changes. The degenerative changes became more pronounced with the increase in the concentration of the genotoxins, as was revealed by severe deformation and nuclear blebbing of the nucleus and other structures. This increase in the dose led to an increase in the apoptotic stimuli, which was confirmed by the apoptotic body near the nucleus (Figure 4a), and nuclear membrane shrinkage was observed in all the treated fish renal cells.

## 4. Discussion

Ultra-morphological analyses through electron microscopy are the best tool to detect various cell intoxication symptoms. Transmission electron microscopy (TEM) produces a two-dimensional image of the cell. It provides the minute details of surface alterations which cannot be observed by the simple light microscope such as an increase or decrease in the number of organelles, nuclear degeneration, loss of cytoplasmic integrity, etc. However, one of the main drawbacks of electron microscopy is that this technique cannot be used to quantify the apoptotic cells [32]. Microscopy is therefore used in the interpretation of the various parameters used to measure the extent of toxicity and contributes to the establishment of measures that aim to prevent environmental contamination, a risk not only to the ecosystem but also to human health [33]. It will support follow-up studies to further elucidate the underlying cellular mechanisms of pesticide toxicity in wildlife. It is pertinent to mention here that the number of fish used in the present study was limited, therefore more work needs to be carried out on true replicates so that there is no room of doubt for the confirmation of the results.

The results of the present study on the ultrastructural changes observed in the kidney cells of common carp, *Cyprinus carpio communis,* exposed to two organophosphate insecticides viz. Phorate and Dimethoate have been compared with the previous findings of other researchers, who observed the toxicological effects of other toxicants in different species. Nandita [34] conducted TEM studies on the kidney of *Ctenopharyngodon iddellus* exposed to an organophosphate monocrotophos (36% *w*/*w* for 15, 30 and 45 days of duration). In the treated fish, several ultrastructural abnormalities were observed in the kidney. The prominent changes included degenerative changes in the nucleus and mitochondria, and cytoplasm in the proximal convoluted tubule showed vacuolization. The number of mitochondria were found to be tremendously increased upon exposure. The degenerative changes were found to be directly linked to the dose concentration of the toxicant as the cellular structures were not clearly visible with a high dosage of the toxicant. Shrinkage of glomerulus, enlargement of Bowman’s capsule, dissociation of epithelial lining and vacuolization of the cytoplasm were also observed in the kidney of fish, *Cirrhinus mrigala*, exposed to metal, lead acetate (chronic toxicity study of 30 days with sub lethal concentrations of 14.1 and 28.2 ppm) [35]. Pesticides cause physiological and biochemical changes in the fish species and greatly influence their behavioural activities. The tubular degeneration was also observed in the kidney of zebrafish, *Danio rerio,* when exposed to a combination of various pesticides (sublethal concentration of 8.4 and 4.2 µg/L of Chloropyrifos 50% + Cypermethrin 5% EC for 7, 14, 21 and 28th day) [36]. Kidney of Asian stinging catfish, *Heteropneutes. fossilis* treated with chlorpyrifos (1/50 of LC_50_ and 1/10 of LC_50_ for a duration of 30 days) also revealed tubular degeneration, vacuolization and loss of glomerular [37]. Similar effects were observed in the kidneys of the brown trout, *Salmo trutta m. fario* when exposed to cadmium and zinc poisoning (4.4 mg/kg and 11 mg/kg, respectively, for a 46-week exposure period [38]. Aluminium also showed its toxic effects on the kidney of Tilapia, *Tilapia zillii*, by causing glomerular shrinkage. This freshwater fish was exposed to three levels of aluminium (25, 50, 100 µg/L) for 96 h [39]. TEM of Almix herbicide at sublethal concentrations of 66.67 mg/L for 30 days on freshwater teleost kidney was found to induce necrosis in the nucleus, severe vacuolization, whorl pattern of ER, and also mitochondrial degeneration [40].

Glomerular podocytes, on the other hand, are highly specialized cells and their most unique features are interdigitated foot processes with filtration slits in between. They are bridged by the slit diaphragm, which plays a major role in establishing the selective permeability of the glomerular filtration barrier [41]. Swollen and fused podocytes and increased infiltration slits observed through TEM in the glomerulus is likely to cause glomerular disease, resulting in failure of the filtration barrier [41]. The ultrastructural examination of the kidney of rainbow trout, *Oncorhynchus mykiss,* exposed to synthetic dyes revealed melanomarcophages increased in number and melanin was more abundant in the cells of treated fish [42].

The kidney thus serves as a major route of excretion of metabolites of xenobiotics [40] and receives the largest proportion of postbranchial blood. It is more likely to undergo histopathological alterations under pesticide stress [14] and, hence, deserves to be examined at cellular level, which is possible only through transmission electron microscopy. The cellular degeneration which occurred in the kidney due to the Phorate and Dimethoate toxicity might have also affected the osmoregulatory function of the fish as the kidney is not only the excretory organ, but also functions as osmoregulatory organ of the fish. The teleost kidney is one of the first organs to be affected by pollutants in water [41]. Thus, the overall ultrastructural changes observed in the kidney of common carp in the present study proved that these organophosphorus insecticides viz. Phorate and Dimethoate are potent genotoxicants which showed their deleterious effects on the osmoregulatory organ of the fish. Previous findings have also proved Phorate to be genotoxic on various other parameters, including the activities of carbohydrate metabolic enzyme, succinate dehydrogenase in common carp [14] and also altered protein content in various tissues of common carp [8]. Phorate also induced pronounced pathological toxicity in the brain of common carp, *Cyprinus carpio* and the extent of damage and degeneration were progressive with the exposure time period and the concentration of Phorate [16]. Phorate also has deleterious effects on humans as it causes cellular damage, as well as changes in DNA structure [7].

The degree of damage to the gill morphology of the Dimethoate-treated fish showed direct correlation to the exposure period, (24, 48 and 96 h when exposed to sub lethal concentration of 0.96 mg/L), while the oxygen consumption and ventilation rate showed a significant decrease during the exposure period to *Cyprinus carpio* [24]. Studies have also shown that Dimethoate can adversely affect the reproductive fitness of male common carps, even at sublethal concentration, both in short-term and long-term exposure, 96 h and 36 days, respectively, when exposed to 0.96 mg/L and 0.48 mg/L [25]. The carp fingerlings exhibit a number of abnormalities in their behaviour when exposed to Dimethoate such as gulping of surface water, uncoordinated movements, tremors and convulsions, excessive mucus secretion, and imbalanced swimming, ending in a collapse to the bottom of the aquarium with wide mouth open [10,11,12]. Histopathological examination of liver of carp exposed to a sublethal concentration of Dimethoate revealed disorganization of hepatocytes, rupture of blood vessels, vacuolization of cytoplasm, pyknotic nuclei and necrosis of the hepatic tissue [23]. *Oncorhynchus mykiss* fish showed remarkable behavioural abnormalities such as loss of balance, erratic swimming, and convulsion and a decrease in protein concentration with the increase in exposure to Dimethoate [19]. Shrinkage of glomerulus, dilation of lumen, vacuolization and tubular degeneration were histopathological changes observed in the kidney of common carp exposed to Dimethoate [21]. Dimethoate-exposed Labeo showed a decrease in protein content [22]. Nortan [27] studied ultrastructural effects of diethylnitrosamine (DENA) and trichloro ethylene (TCE) on the kidney of Japanese medaka, *Oryzias latipes.* Tissues of control and DENA and TCE exposed medaka for 32 weeks were observed by TEM. The kidney of the treated group showed cytotoxic effects along the length of proximal tubule, which were more pronounced in region II, distented proximal tubule, cellular necrosis, membrane fragments, pyknotic nuclei, nuclear membrane disintegration. Marlasca et al., [42] reported degranulation and dilation of endoplasmic reticulum of hepatocytes and renal cells with packed melanosomes in all the developmental stages of rainbow trout exposed to two synthetic dyes (for 30 days) by TEM. Spazier et al., [43] reported severe cellular disintegration of spleen of Anguilla fish exposed to contaminated waters. The cells revealed necrotic changes marked by swelling of the mitochondria. Godar et al., [44] observed apoptosis by dissociation of ribosomes, ER dilation, degeneration of nucleus and chromatin condensation through TEM on inducing the photo toxicity of ultraviolet radiations (UV) of different wave band regions on L5178Y-R murine lymphoma (LYR) cells. Anglade et al., [45] observed apoptotic changes such as loss of contact with surrounding tissues, shrinkage and chromatin condensation in myelinated neurons of substantia nigra in four normal aged humans. Hagar et al., [46] reported ultrastructural anomalies in β-cells of rat pancreas on exposure to dimethoate, as there was a tremendous increase in the number of vacuoles and dilated ERs. The treated group received dimethoate orally via gavage (21 mg/kg) daily for two months. Brajuskovic et al., [47] reported ultrastructural changes such as a reduction in the size of mitochondria of B-chronic lymphocytic leukaemia (B-CLL). The proliferation of smooth endoplasmic reticulum and the presence of several cytosolic electron dense granules were reported in the liver hepatocytes of two the fish species exposed to contaminated waters [48]. The ultrathin sections of the kidney of a malathion-exposed (dose of 27 mg/kg b.wt/day for one month) rat revealed positive signs of degeneration including narrowing of capillary space and deposition of electron dense material in the podocytes and vacuolation of podocyte cytoplasm with fusion of its foot processes [49]. In a nutshell, these examples clearly reveal that our findings are in line with previous studies that suggest that, like other toxicants, these two insecticides, i.e., Phorate and dimethoate, also induce abnormal degenerative changes in cells.

## 5. Conclusions

The results of the present study thus revealed that even the sublethal concentrations of Phorate and Dimethoate induced ultrastructural abnormalities in the kidney of the fish, *Cyprinus carpio communis* and TEM serves as a useful and effective tool for detecting the toxicity of any substance. From the discussion, it is quite evident that the changes or the ultrastructural abnormalities observed during the present study clearly revealed classical morphological changes which occur during apoptosis. Additionally, it was clear that these two widely used insecticides are highly toxic, as was supported by the present TEM study, as well as other previous reports on these two insecticides. Hence, the current study is a proof-of-principle that TEM can be used to examine the cellular impact of pesticide exposure. Since both Phorate and Dimethoate have deleterious effects on fish fauna, their indiscriminate use should be strictly prohibited through legislation.

## Figures and Tables

**Table 1:** Phorate and Dimethoate.

S.NO	NAME	FORMULA	CAS	REG. NO	NATURE
1	Phorate	C_7_H_17_O_2_P_S3_	O, O-diethyl s-[(ethyl thio)methyl] phosphorodithioate	298-02-02	Highly toxic systemic insecticide used as granules. It has fumigant action.
2	Dimethoate	C_5_H_12_O_3_P_S2_	O, O-diethyl s-[2-(methylamino)-2-oxyethyl]phosphorodithioate	60-51-5	It is acutely toxic, has possible links to cancer.

## Data Availability

Not applicable.
